# School Functioning and Educational Aspirations in Adolescents With Social Anxiety—The Young-HUNT3 Study, Norway

**DOI:** 10.3389/fpsyg.2021.727529

**Published:** 2021-10-12

**Authors:** Ingunn Jystad, Tommy Haugan, Ottar Bjerkeset, Erik R. Sund, Jonas Vaag

**Affiliations:** ^1^Faculty of Nursing and Health Science, Nord University, Levanger, Norway; ^2^Department of Public Health and Nursing, Faculty of Medicine and Health Science, Norwegian University of Science and Technology, Trondheim, Norway; ^3^Department of Mental Health, Norwegian University of Science and Technology, Trondheim, Norway; ^4^HUNT Research Centre, Department of Public Health and Nursing, Norwegian University of Science and Technology, Trondheim, Norway; ^5^Levanger Hospital, Nord-Trøndelag Hospital Trust, Levanger, Norway; ^6^Faculty of Social Sciences, Nord University, Levanger, Norway; ^7^Department of Psychology, Norwegian University of Science and Technology, Trondheim, Norway

**Keywords:** social anxiety disorder, SAD, adolescents, educational aspirations, school functioning, bullying, social exclusion, HUNT study

## Abstract

Social anxiety disorder (SAD) typically emerges during childhood or early adolescence and often has long-term effects on several areas of an individual's life, including school and education. The purpose of this study is to examine whether social anxiety is associated with (1) school functioning in terms of behavioral difficulties (hyperactivity and/or attention problems), school dissatisfaction, social exclusion, truancy, and learning difficulties, and (2) educational aspirations (educational level). We use data from the population-based Young-HUNT3 study (2006–2008), where 8,199 Norwegian adolescents participated. Social anxiety is measured both as self-report [the Social Phobia and Anxiety Inventory for Children (SPAI-C)], and as screening information from diagnostic interviews [Anxiety Disorder Interview Schedule for DSM IV: child version (ADIS-C)]. ADIS-C screening positives (*n* = 388) reported higher rates of behavioral difficulties (RR = 1.06), school dissatisfaction (RR = 1.15), social exclusion (RR = 1.24), truancy (RR = 1.05), and learning difficulties (RR = 1.10) compared to screened negatives. Self-reported social anxiety symptoms showed similar patterns. Further, higher mean scores of self-reported social anxiety symptoms and being ADIS-C screening positive were negatively associated with aspirations of higher education (OR = 0.92 and OR = 0.74, respectively). However, as regards to having aspirations for the future (aspirations of higher education and/or aspirations of vocational training), no associations were found. The results indicate that social anxiety in adolescence is related to unfavorable/poorer school functioning and lower tendency of aspirations of higher education, which may have consequences for future educational pathways and later work life.

## Introduction

Social anxiety disorder (SAD) is a common mental disorder across cultures (Stein et al., 2017) and associated with functional impairments affecting both social life and educational attainments (de Lijster et al., 2018; Vilaplana-Pérez et al., 2020). SAD is linked to academic underachievement, in terms of failing a grade in the last year of compulsory school (Vilaplana-Pérez et al., 2020), higher rates of school dropouts (Van Ameringen et al., 2003), and a lower tendency to enter higher education (Kessler, 2003; Vilaplana-Pérez et al., 2020). The negative association between SAD and educational attainment is of particular relevance because low educational level is linked to various unfavorable health outcomes (Krokstad et al., 2002; De Ridder et al., 2012; Myhr et al., 2018).

SAD and social anxiety symptoms at subclinical levels typically emerge during adolescence—a period in life when more time is spent on other arenas than family (Larson and Richards, 1991; Furman and Buhrmester, 1992) and school becomes more challenging, with increasing academic demands. Social anxiety can be measured as a diagnostic phenomenon or expressed along a symptom severity scale (Rapee and Spence, 2004). SAD and social anxiety symptoms are characterized by a marked, persistent fear of social and performance situations in which the individual is exposed to possible, or at least perceived, scrutiny by others (American Psychiatric Association, 2000). Since consistent exposure to social and performance situations is common in schools and especially feared among individuals with social anxiety (Hofmann et al., 1999; Gren-Landell et al., 2009), day-to-day-school life might be especially anxiety-provoking (Mychailyszyn et al., 2010). In a study of Swedish adolescents aged 12–14 yr with self-reported SAD, more than 90% reported impairment in school activities due to their symptoms (Gren-Landell et al., 2009). A Finnish study of 12–17-yr-olds reported lower grade point averages among those with social anxiety and subthreshold symptoms than their healthy peers (Ranta et al., 2009). Finally, a 2-yr longitudinal study of Finnish adolescents aged 15–17 yr using a self-report instrument with a diagnostic cutoff found that SAD predicted slower academic progress among boys but not girls (Ranta et al., 2016).

School functioning includes several arenas and school-related impairment can be linked to academic achievement, as well as social, emotional, and behavioral aspects of the school climate (Mychailyszyn et al., 2010). In a US study of children aged 7–14 yr, individuals with SAD were rated as performing worse academically and being less happy than individuals without a diagnosis (Mychailyszyn et al., 2010). Further, in a non-clinical sample of 45 elementary school children with SAD, Bernstein et al. (2008) reported that condition severity was associated with reduced classroom functioning, such as a greater tendency to experience learning and attention problems and to exhibit poor social and leadership skills. It follows that social anxiety appears to impact several areas of school functioning.

Adolescents' school functioning is closely connected to how they see and plan their future (OECD, 2017; Gutman and Schoon, 2018). Planning an educational career is often rooted in educational aspirations and motivation and ideas of what educational level a person wants to complete. Educational aspirations are strong predictors for achievement in school and later educational attainment (Beal and Crockett, 2010; Rothon et al., 2011; Khattab, 2015). Previous population-based adolescent studies have reported an association between health impairments, psychical and mental health problems, and low educational aspirations (Stevens et al., 1996; Bania et al., 2015; Dobewall et al., 2019). These findings indicate that health challenges represent obstacles to visualizing future potential. In a study of Finnish 12–15-yr-old adolescents, those with good self-rated health were more likely to apply for an academic track. In contrast, those reporting average or poor health were more likely to apply for a vocational track (Dobewall et al., 2019). In the Norwegian Arctic Adolescent Health Study, Bania et al. (2015) reported more intermediate rather than higher-level educational aspirations among adolescents suffering from emotional problems. A Swedish longitudinal study demonstrated that adolescents who reported high academic aspirations had a lower risk of mental health problems 1 yr after, seemingly adding further support for the linkage between health and aspirations (Almroth et al., 2018).

The aim of this study is to investigate whether social anxiety (categorized as self-reported symptoms, being screening positive for SAD or meeting the diagnostic criteria for SAD in a diagnostic interview) is associated with (1) adolescent school functioning, in terms of (a) behavioral difficulties/attention problems, (b) school dissatisfaction, (c) experience of social exclusion, (d) truancy, and (e) learning difficulties, and (2) educational aspirations. We use population data from the social anxiety sub-study in Young-HUNT3 (2006–2008), which consists of both self-reported social anxiety symptoms; the Social Phobia and Anxiety Inventory for Children (SPAI-C), (Beidel et al., 1995), as well as screening information from diagnostic interviews: Anxiety Disorder Interview Schedule for DSM IV: child version (ADIS-C), (Rasmussen and Neumer, 2015).

## Methods

### Sampling and Procedure

This study is based on data from the Young-HUNT3 study (2006–2008), the adolescent part of the third Trøndelag Health Study (HUNT), (Holmen et al., 2013). HUNT study is a cross-sectional population-based health survey conducted in Trøndelag county, Norway, and is one of the world's largest health surveys (Holmen et al., 2003). Further information on HUNT and Young-HUNT study is available elsewhere (Holmen et al., 2003, 2013; Krokstad et al., 2012).

In Young-HUNT3, all adolescents 13–19 yr living in the former Nord Trøndelag county of Norway at the time of the study period, a total of 10,464 adolescents, were invited to participate. The survey consisted of a self-report questionnaire, physical examinations, and clinical interviews. The self-report questionnaire was completed during school hours, whereas the examinations and interviews were carried out ~1 month after. Students absent from school were offered to complete the questionnaire on the interview day, whereas students not attending the interview received the survey by mail (Holmen et al., 2013). A total of 8,199 adolescents (response rate 78.4%) completed the questionnaire, of which 6,610 participated in the social anxiety sub-study (Jystad et al., 2021). See Figure 1 in Jystad et al. (2021) for further details regarding the sampling process.

### Measurements

#### Questionnaire (n = 8,199)

##### Descriptive Variables

Participants were categorized into two *age groups*: 13–15 yr, representing students in lower secondary school, and ≥ 16 yr, representing students in upper secondary school.

*Family economic status* was measured with an item asking whether the adolescent ranged his or her family economic status as worse, better, or equal compared to others.

*School functioning* was measured with 16 items covering topics such as difficulties concentrating in class, appreciation with school participation, and bullying/being bullied, each item rated on an ordinal Likert-scale (1 = never, 2 = sometimes, 3 = often, and 4 = very often). These questions have previously been used to assess school functioning in Young-HUNT (De Ridder et al., 2013; Ranøyen et al., 2014). We wanted to investigate associations between being screening positive for SAD and symptoms of social anxiety, with different measures/patterns of school functioning, and used principal component analysis (PCA) with varimax rotation to identify relevant indices to be used in further analysis. We found that 14 of the 16 items had sufficient factor loadings to cover three different factors; six items covering “behavioral difficulties and/or attention problems” (α = 0.74), related to the ability to pay attention and sit calm during classes; six covering “School enjoyment” (α = 0.73) related to enjoyment in curricular activities; and finally, two items covering “Social exclusion” (α = 0.68), regarding name-calling, and being excluded by peers. In addition, the two remaining items, “truancy” (unexcused school absenteeism) and “learning difficulties” (currently receiving help for learning difficulties), were included in the analyses as single items. The six variables included in “school enjoyment” were reversed in order to look at the association between social anxiety and “school dissatisfaction” (i.e., the lack of school enjoyment).

For the descriptive analyses, a mean score across the items in each factor was computed, a higher score indicating more school functioning problems. For the robust multiple Poisson regression analyses, a sum score for each factor was computed, generating a range of 6–24 for “behavioral difficulties/attention problems” and “school dissatisfaction,” 2–8 for “social exclusion,” and 1–4 for “truancy” and “learning difficulties.”

*Academic aspirations* were assessed by the item “What plans do you have regarding continued studies?” and rated on an ordinal scale “*college or university for 4 yr or more,” “college or university <4 yr,” “other vocational training*,” “*no plans”* and “*don't know,”* allowing for multiple answers. For the descriptive analyses we collapsed it into three categories: the first two were merged to “college or university,” the second was those who reported “other vocational training,” whereas the third category consisted of those who answered “no plans” and “don't know” combined to one: “no plans/don't know.” Furthermore, a hierarchy of the variables was made in such a way that the “highest” alternative became the valid one when multiple answers were given:

1. College or university (highest)
2. Vocational training
3. No plans/don't know (lowest)
  For the multiple logistic regression analyses, we created two dichotomous variables; the first one consisting of 1 = those who had answered “college or university” vs. 0 = those who had *not* answered college or university (as a measure of having plans of higher education); the second consisting of 1 = those who had answered college, university and/or vocational training and 0 = those who had answered only no plans/don't know (as a measure of whether or not having plans for the future).

##### Self-Reported Social Anxiety Symptoms: SPAI-C

The questionnaire included a shortened, six-item version of the original 26-item version of the Social Phobia and Anxiety Inventory for Children (SPAI-C; Beidel et al., 1995), measuring self-reported social anxiety symptoms. SPAI-C is originally a DSM-IV-based self-report measure (Beidel et al., 1995), which has shown satisfying psychometric properties for use among adolescents (Storch et al., 2004). The Norwegian version was translated by Aune and and Hjemdal (2017), and the six items included in Young-HUNT were chosen based on factor analysis (Ranøyen et al., 2014). The items were all rated on a 5-point Likert scale, ranging from 1 = never to 5 = always. A mean SPAI-C score (Cronbach's alpha = 0.84) was calculated; higher mean scores indicating higher levels of symptoms.

##### Anxiety and Depression Symptoms (SCL-5)

To assess general symptoms of anxiety and depression during the past 2 weeks, a shortened five-item-version of the original 25-item Symptom Check List (SCL), (Derogatis et al., 1974), was used. Each item had four response options, from 1 = not bothered to 4 = very bothered. The five-item version (SCL-5) has shown adequate reliability and validity (Strand et al., 2003). A mean SCL-score was calculated across the five items (Cronbach's alpha = 0.83), with higher mean scores indicating higher symptom levels.

#### SAD Screening/Anxiety Disorders Interview Schedule for DSM IV: Children's Version (n = 6,610)

In addition to self-report information from the questionnaire, Young-HUNT3 included a social anxiety sub-study using the Anxiety Disorders Interview Schedule for DSM IV: Child Version (ADIS-C). ADIS-C is a diagnostic interview based on the DSM-IV criteria used to diagnose anxiety disorders and other mental disorders among children and adolescents (Rasmussen and Neumer, 2015). The social anxiety sub-study included an initial screening phase involving three items from the social anxiety part of ADIS-C and a complete diagnostic ADIS-C interview of the screening positive adolescents. Two municipalities did not participate in this part of Young-HUNT3, which resulted in the exclusion of *n* = 1,589 participants. Of the remaining *n* = 6,610 participating adolescents, a total of *n* = 388 (5.9%) answered yes to at least one of the questions and were considered as screening positive (SP). Adolescents who answered no to all three questions (*n* = 6,222) were considered as screening negative (SN), (Jystad et al., 2021). Further details regarding the social anxiety sub-study are available in Jystad et al. (2021).

### Groups

In the statistical analyses, the study sample was divided into the following subgroups:

1. ADIS-C SN (*n* = 6,222; answered no to all three screening questions)

2. ADIS-C SP (*n* = 388; answered yes to one or more SAD screening questions)

As a part of the social anxiety sub-study in Young-HUNT3, the adolescents who screened positive were invited to a complete diagnostic ADIS-C interview performed by specially trained nurses. A total of *n* = 212 (response rate 54.6%) participated, of which a SAD diagnose was indicated in *n* = 106 (50% of the interview objects), which generated the following three SP subgroups:

- ADIS-C SP, SAD confirmed by ADIS-C interview (*n* = 106) (SAD)
- ADIS-C SP, SAD not confirmed by ADIS-C interview with trained nurses (*n* = 106) (SPNOSAD)
- ADIS-C SP that did not attend the interview (*n* = 176) (NMI).

### Statistics

Stata version 15.1 (StataCorp, 2017) was used for data management and statistical analyses.

First, a descriptive analysis was performed for the study groups with respect to mean scores for the three factors of school functioning (behavioral difficulties and/or attention problems, school dissatisfaction, and social exclusion) as well as the two single items (learning problems and truancy/school absenteeism), for educational aspirations, and for the additional descriptive variables: sex, age groups, age mean, age distribution, family economic status, self-reported social anxiety symptoms (mean score SPAI-C), general anxiety and depression symptoms (mean score SCL-5).

Second, in multiple Poisson regression analysis, requesting robust standard errors, associations were estimated between the SP group (*n* = 388) and five factors of school functioning: sum score of behavioral difficulties/attention problems, sum score of school dissatisfaction, and sum score of social exclusion using SN group as a reference. The same procedure was performed for the two single items of school functioning (learning difficulties and truancy). Further, the variables of school functioning were investigated using mean SPAI-C scores (continuous variable), (“SPAI-C score”), as predictor variables in the robust multiple Poisson regression model, involving the total sample who answered the self-report questionnaire (*n* = 8,199). The same was performed with a mean score of SCL-5 (“SCL-5 score”) as the predictor (*n* = 8,199). Results are reported as rate ratios (RR) along with 95% confidence intervals (95% CI).

Finally, multiple logistic regression analysis was performed to estimate the odds ratio (OR) for the association between the ADIS-C SP group and aspirations for further education, using the ADIS-C SN group as the reference group (*n* = 6,610). The same procedure was performed using mean scores of (1) SPAI-C and (2) SCL-5 as predictors, involving the total sample (*n* = 8,199). The analyses for SPAI-C and SCL-5 as predictors were then repeated and performed separately for students in lower secondary school (13–15 years of age) and upper secondary school (≥16 years of age). Results are reported as odds ratios (OR) with 95% confidence intervals (95% CI). All analyses were adjusted for sex, age (measured on a continuous scale), and perception of family economic status.

Finally, multiple logistic regression analysis was performed to estimate the odds ratio (OR) for the association between the ADIS-C SP group and aspirations for further education, using the ADIS-C SN group as the reference group (*n* = 6,610). The same procedure was performed using mean scores of (1) SPAI-C and (2) SCL-5 as predictors, involving the total sample (*n* = 8,199). The analyses for SPAI-C and SCL-5 as predictors were then repeated and performed separately for students in lower secondary school (13–15 years of age) and upper secondary school (≥16 years of age). Results are reported as odds ratios (OR) with 95% confidence intervals (95% CI). All analyses were adjusted for sex, age (measured on a continuous scale), and perception of family economic status.

Analyses of the SP subgroups were also performed, the results are presented in Supplementary Tables 1–3.

### Ethics

Participation in the Young-HUNT3 study was voluntary. Informed written consent was signed by the participants. For participants under the age of 16, signed permission from the parents was necessary (Holmen et al., 2013). The research protocol was approved by Regional Committees for Medical and Health Research Ethics (REK).

## Results

### Descriptive Characteristics

Table 1 shows descriptive data for the study groups. A total of *n* = 388 adolescents were in the SP group − 267 (68.8%) girls and 121 (31.2%) boys. A total of *n* = 6,222 of the adolescents were in the SN group − 3,063 (49.2%) girls and 3,159 (50.8%) boys. The SP group reported higher mean scores for behavioral difficulties/attention problems (SP: 1.71, SN: 1.61), school dissatisfaction (SP: 2.46, SN: 2.12), social exclusion (SP: 1.53, SN: 1.24), truancy (SP: 1.35, SN: 1.25), and learning difficulties (SP: 1.37, SN: 1.27), compared to the SN group. The same tendencies were observed for the three SP subgroups within the SP group (see Supplementary Table 1 in Supplementary). In addition, the SAD subgroup reported the highest mean score for social exclusion (1.64) (see Supplementary Table 1 in Supplementary).

**Table 1:** Descriptive characteristics of adolescents in Young-HUNT3 categorized/identified as ADIS-C screening negative (*n* = 6,222) and ADIS-C screening positive (*n* = 388).

	**SAD (ADIS-C) screening negatives**	**SAD (ADIS-C) screening positives**
**Sex** ***n*** **(%)**		
Girls	3,063 (49.23)	267 (68.81)
Boys	3,159 (50.77)	121 (31.19)
**Age mean (SD)**	15.97 (1.70)	16.12 (1.90)
**Age distribution** ***n*** **(%)**		
13–15 years	3,176 (51.04)	195 (50.26)
≥16 years	3,046 (48.96)	193 (49.74)
**Family economic status** ***n*** **(%)**		
Worse	497 (8.46)	57 (15.92)
Equal	4,311 (73.42)	250 (69.83)
Better	1,064 (18.12)	51 (14.25)
**Mean all social anxiety items (SPAI-C) (SD)**	1.86 (0.67)	2.82 (0.87)
**Mean anxiety and depression items (SCL-5) (SD)**	1.47 (0.52)	2.01 (0.72)
**Mean school functioning (SD)**		
Behavioral difficulties/attention problems (1–4)	1.61 (0.43)	1.71 (0.41)
School dissatisfaction (1–4)	2.12 (0.52)	2.46 (0.51)
Social exclusion/bullying (1–4)	1.24 (0.45)	1.53 (0.67)
Truancy (single item) (1–4)	1.25 (0.49)	1.35 (0.57)
Learning difficulties (single item) (1–4)	1.27 (0.67)	1.37 (0.79)
**Educational aspirations** ***n*** **(%)**		
No plans/don't know	2,383 (41.79)	164 (45.81)
Vocational training	1,082 (18.97)	72 (20.11)
University	2,238 (39.24)	122 (34.08)

Missing values: for family economic status, values were missing for *n* = 380/5.7% of the 6,610 participants in social anxiety sub-study of Young-HUNT3, *n* = 30/7.7% of the 388 screening positives. Among the 6,610, missing values for SPAI-C ranged between *n* = 167/2.5%, and *n* = 187/2.8% across the six items. The summed mean score missed values for *n* = 261/3.9%, *n* = 19/4.9% of the 388 screening positives. Among the 6,610, missing values for SCL-5 ranged between *n* = 155/2.3%, and *n* = 165/2.5% across the six items. The summed mean score missed values for *n* = 209/3.2%, *n* = 16/4.1% of the 388 screening positives.

Among the 6,610 participants, mean scores of behavioral difficulties/attention problems had missing values for *n* = 576/8.7%, and *n* = 40/10.3% of the 388 screening positives, mean scores of school dissatisfaction: *n* = 555/8.4% and *n* = 46/11.9%, and mean scores of social exclusion: *n* = 483/7.3% and *n* =36/9.3%. For the single items, missing values were *n* = 420/6.4% and *n* = 33/8.5% (truancy), and *n* = 671/10.2% and *n* = 48/12.4% (learning difficulties). For educational aspirations, variables were missing for *n* = 549/8.3% of the 6,610 participants, *n* = 30/7.7% of the 388 screening positives. More detailed descriptions of the sample described in this table can be found in Jystad et al. (2021).

A lower proportion of the SP group reported aspirations of going to university compared to the SN group (SP: 34.1%, SN: 39.2%), and a higher proportion of the SP group answered no plans/don't know (SP: 45.8%, SN: 41.8%). This was also partly observed for the three SP subgroups (see Supplementary Material). Regarding age differences, a higher proportion of adolescents under the age of 16 answered no plans/don't know (48.6%) compared to adolescents over the age of 16 (35.4%). For remaining descriptive characteristics, see Table 1.

### Social Anxiety and School Functioning

Table 2 shows associations expressed as RR between (1) ADIS-C SP group, (2) self-reported social anxiety symptoms on a continuous scale (SPAI-C), (3) self-reported mental distress (SCL-5), and the five indicators of school functioning (behavioral difficulties/attention problems, school dissatisfaction, social exclusion, truancy and learning difficulties). After adjusting for sex, age, and family economic status, the SP group reported a 24% higher rate of social exclusion compared to the SN group [RR: 1.24, CI: (1.19–1.30)]. Similarly, compared to the SN group, the SP group reported higher rates of behavioral difficulties/attention problems [RR: 1.06, CI: (1.04–1.09)], school dissatisfaction [RR: 1.15, CI: (1.13–1.18)], truancy [RR: 1.05, CI: (1.01–1.10)], and learning difficulties [RR: 1.10, CI: (1.03–1.17)]. Similar directions of associations were observed for the SP subgroups (see Supplementary Table 2) and for social anxiety symptoms measured on a continuum (SPAI-C); a one-unit increase in the mean score of social anxiety symptoms was associated with an 8% increase in the rate of behavior difficulties/attention problems, a 9% increase in school dissatisfaction, a 15% increase in experience of social exclusion, a 6% increase in score of truancy, and a 5% increase in learning difficulties. Similar directions of associations were observed between SCL-5 and indicators of school functioning.

**Table 2:** Associations^*^ between symptoms of SAD and mental distress and indicators of school functioning among adolescents.

	**Behavioral difficulties/attention problems Range: 6–24**		**Schooldissatisfaction Range: 6–24**		**Social exclusion/bullying Range: 2–8**		**Truancy** **Range: 1–4**		**Learning difficulties** **Range: 1–4**	
	**RR**	**95% CI**	**RR**	**95% CI**	**RR**	**95% CI**	**RR**	**95% CI**	**RR**	**95% CI**
*Screening status^a^*	(*n* = 5,983)		(*n* = 6,003)		(*n* = 6,074)		(*n* = 6,131)		(*n* = 5,887)	
**Screening ADIS-C negative**	1 (reference)		1 (reference)		1 (reference)		1 (reference)		1 (reference)	
**Screening ADIS-C positive**	**1.06**	1.04**–**1.09	**1.15**	1.13**–**1.18	**1.24**	1.19**–**1.30	**1.05**	1.01**–**1.10	**1.10**	1.03**–**1.17
*Self-report^b^ social anxiety symptoms*	(*n* = 7,116)		(*n* = 7,206)		(*n* = 7,276)		(*n* = 7,352)		(*n* = 7,062)	
**SPAI-C score**	**1.08**	1.07**–**1.09	**1.09**	1.08**–**1.10	**1.15**	1.13**–**1.16	**1.06**	1.04**–**1.07	**1.05**	1.03**–**1.07
*Self-report^b^ mental distress*	(*n* = 7,189)		(*n* = 7,226)		(*n* = 7,302)		(*n* = 7,376)		(*n* = 7,079)	
**SCL-5 score**	**1.17**	1.16**–**1.19	**1.11**	1.10**–**1.13	**1.22**	1.19**–**1.24	**1.14**	1.12**–**1.16	1.01	0.99**–**1.04

*Rate ratio (RR) and 95% confidence interval (95% CI)*.

*Values where CI do not cross 1 are put in bold*.

* *Adjusted for sex, age and family economic status*.

a *Missing values for school functioning and adjustment variables among ADIS-C screening negatives (n = 6,222), and ADIS-C screening positives (n = 388): see Table 1*.

b *Missing values among the total of n = 8,199 participants answering self-report questionnaire in Young-HUNT3: sum scores of behavioral difficulties/attention problems had missing values for n = 834/10.2%, sum scores of school dissatisfaction for n = 794/9.7%, and sum scores of social exclusion for n = 707/8.6%. For the single items, missing values were n = 615/7.5/% (truancy), and n = 930/11.3% (learning difficulties). Missing values for family economic status were n = 563/6.9%*.

### Social Anxiety and Educational Aspirations

Table 3 shows the associations expressed as ORs adjusted for sex, age, and perception of family economic status, between the (1) ADIS-C SP group, (2) self-reported social anxiety on a continuous scale and (3) self-reported mental distress, and aspirations of higher education (university). Being in the SP group (*n* = 388) was associated with a 26% reduction in odds of having aspirations of going to university/higher education. A lower tendency was also observed for the NMI subgroup but not for SPNOSAD and SAD subgroups (see Supplementary Table 3). No associations between the SP group and aspirations for the future were found.

**Table 3:** Associations between various degrees of social anxiety symptoms and mental distress, and aspirations of higher education and aspirations for the future.

	**Aspirations of higher education**		**Aspirations for the future**	
	**Adjusted OR**	**95% CI**	**Adjusted OR**	**95% CI**
Screening status^*^	(*n* = 5,725)		(*n* = 5,725)	
**Screening ADIS C neg**	1 (reference)		1 (reference)	
**Screening ADIS-C pos**	**0.74^a^**	0.58–0.94	0.87^a^	0.69–1.09
Self-report^**^	(*n* = 6,886)		(*n* = 6,886)	
*social anxiety symptoms*				
**SPAI-C score**				
All	**0.92^a^**	0.85–0.99	0.95^a^	0.88–1.02
Age group <16 yr	1.01^b^	0.91–1.11	1.04^b^	0.94–1.14
Age group ≥ 16 yr	**0.86^b^**	0.77–0.95	**0.87^b^**	0.79–0.97
Self-report^**^	(*n* = 6,913)		(*n* = 6,913)	
*mental distress*				
**SCL-5 score**				
All	**1.18^a^**	1.08–1.30	**1.15^a^**	1.05–1.27
Age group <16 yr	**1.41^b^**	1.23–1.61	**1.39^b^**	1.22–1.59
Age group ≥ 16 yr	1.05^b^	0.92–1.20	0.99^b^	0.86–1.14

*Odds ratio (OR) and 95% confidence interval (95% CI)*.

*Values where CI do not cross 1 are put in bold*.

a *Adjusted for sex, age and family economic status*.

b *Adjusted for sex, and family economic status*.

* *Missing values for educational aspirations and adjustment variables among ADIS-C screening negatives (n = 6,222) and ADIS-C screening positives (n = 388): see Table 1*.

** *Missing values among the total of n = 8,199 participants answering self-report questionnaire in Young-HUNT3: educational aspirations had missing values for n = 653/8.0%. Missing values for family economic status were n = 563/6.9%*.

Table 3 also shows associations, adjusted for sex, age, and perception of family economic status, between (1) self-reported social anxiety symptoms (SPAI-C) and (2) self-reported mental distress (SCL-5), both as continuous scores, and aspirations of higher education (university). For adolescents ≥ 16 yr of age, a one-unit increase in mean SPAI-C was associated with a 14% reduction in odds of having aspirations of the university. For adolescents <16 yr, no associations were found. For SCL-5, the case is the opposite; in the younger age group (<16 yr), a one-unit increase in mean SCL-5 score was associated with a 41% *increase* in odds for having aspirations of higher education. In contrast, no association was found for adolescents 16 yr or older.

## Discussion

This large population-based study of >6,000 Norwegian adolescents shows that social anxiety (measured by self-reported symptoms, ADIS-C screening questions and diagnosed by a complete ADIS-C interview based on DSM-IV criteria) was associated with lower school functioning as indicated by higher reports of behavioral difficulties/attention problems, school dissatisfaction, social exclusion, truancy and learning difficulties. Overall, results from our study lend further support to previous research indicating that individuals with social anxiety struggle to cope in the school situation (Davidson et al., 1993; Essau et al., 1999; Wittchen et al., 1999; Chartier et al., 2001; Bernstein et al., 2008; Ranta et al., 2009; Mychailyszyn et al., 2010; Ranøyen et al., 2014). Furthermore, among adolescents with social anxiety, we found lower tendency of aspirations of higher education, in particular among students attending upper secondary school. Our findings are comparable with previous studies reporting associations between poor mental health and low educational aspirations in general (Rothon et al., 2011; Bania et al., 2015; Dobewall et al., 2019).

### School Functioning and Educational Aspirations

The association between social anxiety and increased risk of behavioral difficulties, attention problems, or both may reflect that individuals with social anxiety struggle in the classroom situation, which requires students to be calm, concentrated, and pay attention to the teacher. This is in accordance with previous findings among students with social anxiety, both among adolescents (Davidson et al., 1993) and children (Bernstein et al., 2008; Van Roy et al., 2009). Higher mean scores of self-reported social anxiety symptoms (SPAI-C) were positively associated with behavioral difficulties/attention problems. A study based on parent and teacher information also found that severity of social anxiety was associated with greater attention problems (Bernstein et al., 2008). It is likely that attention problems are derived from distractions caused by the fear and anxiety they experience in the classroom situation (Bernstein et al., 2008).

Social anxiety was positively associated with school dissatisfaction. Although we studied a somewhat older cohort, this is comparable with findings from an American study of school functioning of children and adolescents, where pupils with SAD were rated as less happy compared to their healthy peers (Mychailyszyn et al., 2010). It is also in line with findings from a retrospective clinical study of 201 adults with anxiety disorders, where 90 of the patients (45%) reported not enjoying school when they were children (Van Ameringen et al., 2003). In a review regarding school absenteeism in youth, boredom and lack of interest in school were reported as important risk factors for school dropout (Kearney, 2008). However, in the study by Van Ameringen et al. (2003), the majority of the sample described typical symptoms of social anxiety—nervousness in the school situation and discomfort speaking in front of the class—as the main reasons for not enjoying and dropping out of school or both. This may indicate that lack of school enjoyment among students with social anxiety is linked to symptom burden in the school situation, rather than boredom.

Social anxiety was positively associated with social exclusion. Our results support previous evidence for the tendency of individuals with social anxiety to report having fewer friends (Erath et al., 2010), having trouble making new friends (Bernstein et al., 2008), as well as experiencing poor support from friends (La Greca and Lopez, 1998). In light of social exclusion being defined as one type of bullying (Smith et al., 2002), findings also support previous evidence of the link between social anxiety and peer victimization/bullying (Ranta et al., 2009; Gren-Landell et al., 2011; Acquah et al., 2016; de Lijster et al., 2018; Chiu et al., 2021). Our results are worrisome, considering that poor peer experiences have been linked to school dropout later on, adult adjustment problems (Parker and Asher, 1987), as well as an elevated risk for social anxiety symptoms later in life (Chiu et al., 2021).

Not surprisingly, social anxiety was also positively associated with truancy. Avoidance of situations generating anxiety is one of the main aspects of the condition. The principle of mandatory schooling, however, demands the pupil to participate in activities typically feared, and therefore, truancy may be the consequence (Ingul, 2014). A positive association between self-reported social anxiety symptoms and school absence has previously been reported among Norwegian adolescents (Ingul et al., 2012) whereas an association between truancy and screening questions was indicated in a German survey investigating school absenteeism among 1,359 high school students, where there was a higher percentage among those reporting non-attendance during the past 7 days that reported to feel anxiety or nervousness in unfamiliar situations, compared to the attendant students (35% vs. 22%), (Pflug and Schneider, 2016). In a clinical study of American children diagnosed with SAD, 10% reported that they regularly refused to attend school (Beidel et al., 1999), demonstrating that the link between social anxiety and school absence is present also among younger pupils. Finally, in Van Ameringen et al. (2003) study of 201 adults with anxiety disorders, 49% had dropped out of school, in which the majority suffered from SAD. In spite of our results and the literature linking social anxiety to school absences, it is important to keep in mind that the causes of school absences are various and influenced by a range of factors (Kearney, 2008). Lastly, it is worth mentioning that anxiety-based school absenteeism most usually is referred to as “school refusal” (Kearney, 2008). In contrast, the term “truancy” refers to unexcused, illegal, and problematic school absenteeism, which most often is used when speaking of school absence related to externalizing problems (Kearney and Silverman, 1996; Egger et al., 2003). However, the terms are used interchangeably. Our choice of using “truancy” is based on the Norwegian term “skulk” used in the questionnaire, which usually refers to an unexcused version of school absenteeism.

Our results revealed a weak association between social anxiety and the degree of learning difficulties. However, it is challenging to determine whether social anxiety leads to learning difficulties or if the case is the other way around (Ranøyen et al., 2014). A possible explanation could be that the constant fear of school-related social and performance activities in the classroom requires so much energy that the individual is hindered in focusing on learning. On the other hand, it is possible that having learning difficulties lead to fear of being scrutinized in the classroom. However, neither of these two causal directions are mutually exclusive.

Regarding educational aspirations, social anxiety was associated with lower odds of reporting aspirations of higher education. Considering that the main characteristics of SAD and social anxiety are fear and avoidance of social and performance situations (American Psychiatric Association, 2000), which are common in school and educational settings, a strategy of preventing future discomfort by not planning to attend higher education, is reasonable. Thus, the choice of career may be motivated by the hope of avoiding uncomfortable situations in adult life. In addition, it is worth reflecting upon the fact that social anxiety has been linked with negative self-imagery (Chapman et al., 2020), probably leading to less confidence in own abilities and aspirations matching only what is anticipated as manageable. To the best of our knowledge, there is a lack of previous studies of educational aspirations among adolescents with social anxiety, and therefore, exact comparisons of our results to other studies are challenging. However, compared to poor mental health in general, our findings are in accordance with Rothon et al. (2011) study of British children and adolescents who reported lower educational aspirations among those reporting psychological stress and to a Norwegian study that reported higher aspirations among students reporting less hyperactivity and attention problems, and more intermediate rather than high aspirations among adolescents with emotional problems (Bania et al., 2015). On the other hand, our results contrast a Slovak adolescent study that did not show any association between adolescent's self-rated health and educational aspirations, however not specified as either physical or mental health (Madarasova Geckova et al., 2010).

Even though there are associations between social anxiety (both measured as screening positive/negative and on a continuous scale) and *plans for higher education*, these associations are less clear when looking at *plans for the future*. The association between social anxiety and *plans for the future* seems to become clearer after the age of 16. When looking at self-reported social anxiety symptoms (SPAI-C), the negative association with educational aspirations was only found among pupils in upper secondary school. A possible explanation for the lack of association among students in lower secondary school could be that they have not devoted much attention to future plans yet. The fact that a higher proportion of adolescents under the age of 16 answered no plans/don't know (48.6% vs. 35.4%) may reflect this issue. A high proportion of undecided individuals were also found by Bania et al. (2015) which studied a sample of 15-and 16-yr-olds. Interestingly, the results showed the opposite for general anxiety and depression symptoms (SCL-5), where increasing symptom levels were associated with aspirations of higher education only for individuals in the younger age group.

Interestingly, the lowest odds of having aspirations of higher education and future educational plans, were found among ADIS-C SP that did not meet to interview. This subgroup with regard to symptom load and sociodemographic characteristics is further discussed in Jystad et al. (2021).

Several authors have adjusted for socioeconomic background when studying associations between health impairments and educational aspirations (Bania et al., 2015; Almroth et al., 2018; Dobewall et al., 2019), and all our analyses were adjusted for subjective perception of family economic status, in addition to sex and age. Norway is a welfare state, and education, including university, is free. However, previous studies have shown that parent's education and employment seem to affect their children's probability of completing upper secondary school and choice of educational track, both in Norway and other Nordic welfare states like Finland (Koivusilta et al., 2013; Bania et al., 2015; Kallio et al., 2016; Haugan and Myhr, 2019), demonstrating that choice of career is affected by socioeconomic background.

To sum up, our results lend further support to previous findings indicating that mental health problems may represent obstacles to realize one's own full potential early in an educational pathway and contribute new information by showing the association for individuals with social anxiety, specifically.

### Strengths and Limitations

Young-HUNT3 is a large population-based study inviting all adolescents aged 12–19 yr in former Nord Trøndelag county of Norway. In the case of social anxiety, the use of a population sample rather than a clinical sample is beneficial; due to shyness, avoidant behavior, and fear of authority figures, individuals with social anxiety tend not to seek help for their problems (Wittchen et al., 1999; Grant et al., 2005). In addition, the study is based on both self-reported social anxiety symptoms (SPAI-C) as well as screening information based on interviews performed by specially trained nurses (ADIS-C).

As this study was based on cross-sectional data, there are substantial limitations when interpreting the associations found, yielding it important to investigate this relationship further using prospective data. Another limitation may be the operationalization of school functioning. The five items of school functioning that we used were based on three indices (behavior difficulties/attention problems, school enjoyment, and social exclusion) retrieved from principal component analysis based on 14 items and two single items that covered other important areas of school functioning (truancy and learning difficulties). Although this instrument has been used in previous studies (De Ridder et al., 2013; Ranøyen et al., 2014), the instrument lacks proper validation.

Also, in our multiple regression analysis, when measuring associations between social anxiety and educational aspirations, we adjusted for sex, age, and family economic status. Due to previous report of higher average marks being associated with higher educational aspirations (Bania et al., 2015), as well as a study controlling for academic achievement when measuring associations between health and aspirations (Dobewall et al., 2019), it could be argued that the indications of school functioning should be adjusted for. If school functioning were to be an important covariate, it is possible that we have overestimated the association between social anxiety and educational aspirations. Our decision for not adjusting for school functioning in our main analyses is based on the assumption of school functioning primarily being a consequence of social anxiety. Despite this, one cannot omit the possibility of school functioning and social anxiety working bi-directionally, which has been found for peer victimization and social anxiety among boys (Ranta et al., 2013). Last, our study did not contain information regarding parental expectations, parental educational status, or family structure, which in other studies have shown to be associated with adolescent's future educational plans (Garg et al., 2007; Madarasova Geckova et al., 2010; Christofides et al., 2015), and theoretically also could have an impact on social anxiety status. Therefore, one needs to carefully interpret the results.

## Conclusion

Our results indicate that social anxiety, measured as self-reported social anxiety symptoms and SAD screening interview, is related to lower school functioning in terms of higher tendency of behavior difficulties/attention problems, social exclusion, truancy, learning difficulties. In addition to lack of enjoyment, as well as aspirations of higher education, among Norwegian adolescents. Further studies should investigate prospectively the associations between social anxiety disorder in adolescence and later educational and professional achievements using registry data. In addition, our findings may have valuable implications for teachers and other school professionals that should be observant of social anxiety among pupils showing behavioral difficulties and/or attention problems and truancy. And finally, considering previous evidence of school engagement being positively associated with life satisfaction (Lewis et al., 2011), our findings of school dissatisfaction among adolescents with social anxiety underscores the importance of detection of social anxiety at an early stage, making interventions possible.

## Data Availability Statement

The Trøndelag Health Study (HUNT) has invited persons aged 13–100 yr to four surveys between 1984 and 2019. Comprehensive data from more than 140,000 persons having participated at least once and biological material from 78,000 persons are collected. The data are stored in HUNT databank and biological material in HUNT biobank. HUNT Research Center has permission from the Norwegian Data Inspectorate to store and handle these data. The key identification in the data base is the personal identification number given to all Norwegians at birth or immigration, whilst de-identified data are sent to researchers upon approval of a research protocol by the Regional Ethical Committee and HUNT Research Center. To protect participants' privacy, HUNT Research Center aims to limit storage of data outside HUNT databank, and cannot deposit data in open repositories. HUNT databank has precise information on all data exported to different projects and are able to reproduce these on request. There are no restrictions regarding data export given approval of applications to HUNT Research Center. For more information see: http://www.ntnu.edu/hunt/data. Requests to access the datasets should be directed to http://www.ntnu.edu/hunt/data.

## Ethics Statement

The studies involving human participants were reviewed and approved by Regional Committee for Medical and Health Research Ethics. Written informed consent to participate in this study was provided by the participants' legal guardian/next of kin.

## Author Contributions

All authors participated in planning the outlines of the study, including strategy for data usage, and analysis. IJ conducted the analysis with assistance from ES and JV. IJ wrote the outlines of the manuscript, with input and support from all authors, did the majority of work on all aspects of writing introduction, methodology, results and discussion, with support, and contribution from all authors. All authors contributed to the article and approved the submitted version.

## Conflict of Interest

The authors declare that the research was conducted in the absence of any commercial or financial relationships that could be construed as a potential conflict of interest.

## Publisher's Note

All claims expressed in this article are solely those of the authors and do not necessarily represent those of their affiliated organizations, or those of the publisher, the editors and the reviewers. Any product that may be evaluated in this article, or claim that may be made by its manufacturer, is not guaranteed or endorsed by the publisher.
