# Corrigendum: School functioning and educational aspirations in adolescents with social anxiety—The Young-HUNT3 study, Norway

**DOI:** 10.3389/fpsyg.2023.1228198

**Published:** 2023-06-30

**Authors:** Ingunn Jystad, Tommy Haugan, Ottar Bjerkeset, Erik R. Sund, Jonas Vaag

**Affiliations:** ^1^Faculty of Nursing and Health Science, Nord University, Levanger, Norway; ^2^Department of Public Health and Nursing, Faculty of Medicine and Health Science, Norwegian University of Science and Technology, Trondheim, Norway; ^3^Department of Mental Health, Norwegian University of Science and Technology, Trondheim, Norway; ^4^HUNT Research Centre, Department of Public Health and Nursing, Norwegian University of Science and Technology, Trondheim, Norway; ^5^Levanger Hospital, Nord-Trøndelag Hospital Trust, Levanger, Norway; ^6^Faculty of Social Sciences, Nord University, Levanger, Norway; ^7^Department of Psychology, Norwegian University of Science and Technology, Trondheim, Norway

**Keywords:** social anxiety disorder, SAD, adolescents, educational aspirations, school functioning, bullying, social exclusion, HUNT study

In the published article, there was an error in Table 1 as published. In the rows “Mean all social anxiety items (SPAI-C), (sd),” “Mean anxiety and depression items (SCL-5) (sd),” “Behavioral difficulties/attention problems (1–4),” “School dissatisfaction (1–4),” “Social exclusion/bullying (1–4),” “Truancy (single item) (1–4),” and “Learning difficulties (single item) (1–4),” the mean values were erroneously reported with standard errors (SE) instead of standard deviations (SD). The corrected Table 1 and its caption appear below.

**Table 1:** Descriptive characteristics of adolescents in YoungHUNT3 categorized/identified as ADIS-C screening negative (*n* = 6,222) and ADIS-C screening positive (*n* = 388).

	**SAD (ADIS-C) screening negatives**	**SAD (ADIS-C) screening positives**
**Sex** ***n*** **(%)**		
Girls	3,063 (49.23)	267 (68.81)
Boys	3,159 (50.77)	121 (31.19)
**Age mean (SD)**	15.97 (1.70)	16.12 (1.90)
**Age distribution** ***n*** **(%)**		
13–15 years	3,176 (51.04)	195 (50.26)
≥16 years	3,046 (48.96)	193 (49.74)
**Family economic status** ***n*** **(%)**		
Worse	497 (8.46)	57 (15.92)
Equal	4,311 (73.42)	250 (69.83)
Better	1,064 (18.12)	51 (14.25)
**Mean all social anxiety items (SPAI-C) (SD)**	1.86 (0.67)	2.82 (0.87)
**Mean anxiety and depression items (SCL-5) (SD)**	1.47 (0.52)	2.01 (0.72)
**Mean school functioning (SD)**		
Behavioral difficulties/attention problems (1–4)	1.61 (0.43)	1.71 (0.41)
School dissatisfaction (1–4)	2.12 (0.52)	2.46 (0.51)
Social exclusion/bullying (1–4)	1.24 (0.45)	1.53 (0.67)
Truancy (single item) (1–4)	1.25 (0.49)	1.35 (0.57)
Learning difficulties (single item) (1–4)	1.27 (0.67)	1.37 (0.79)
**Educational aspirations** ***n*** **(%)**		
No plans/don't know	2,383 (41.79)	164 (45.81)
Vocational training	1,082 (18.97)	72 (20.11)
University	2,238 (39.24)	122 (34.08)

Missing values: for family economic status, values were missing for *n* = 380/5.7% of the 6,610 participants in social anxiety sub-study of Young-HUNT3, *n* = 30/7.7% of the 388 screening positives. Among the 6,610, missing values for SPAI-C ranged between *n* = 167/2.5%, and *n* = 187/2.8% across the six items. The summed mean score missed values for *n* = 261/3.9%, *n* = 19/4.9% of the 388 screening positives. Among the 6,610, missing values for SCL-5 ranged between *n* = 155/2.3%, and *n* = 165/2.5% across the six items. The summed mean score missed values for *n* = 209/3.2%, *n* = 16/4.1% of the 388 screening positives.

Among the 6,610 participants, mean scores of behavioral difficulties/attention problems had missing values for *n* = 576/8.7%, and *n* = 40/10.3% of the 388 screening positives, mean scores of school dissatisfaction: *n* = 555/8.4% and *n* = 46/11.9%, and mean scores of social exclusion: *n* = 483/7.3% and *n* =36/9.3%. For the single items, missing values were *n* = 420/6.4% and *n* = 33/8.5% (truancy), and *n* = 671/10.2% and *n* = 48/12.4% (learning difficulties). For educational aspirations, variables were missing for *n* = 549/8.3% of the 6,610 participants, *n* = 30/7.7% of the 388 screening positives. More detailed descriptions of the sample described in this table can be found in Jystad et al. (2021).

In the published article, there were also errors in the text. In the descriptions of the statistical methods, a sentence was erroneously placed in paragraph 3 in the description of Poisson regression analyses. It should be placed in paragraph 4 in the description of multiple logistic regression analyses.

A correction has been made to *Methods, Statistics, Paragraphs 3* and *4*. The corrected paragraphs are shown below.

Second, in multiple Poisson regression analysis, requesting robust standard errors, associations were estimated between the SP group (*n* = 388) and five factors of school functioning: sum score of behavioral difficulties/attention problems, sum score of school dissatisfaction, and sum score of social exclusion using SN group as a reference. The same procedure was performed for the two single items of school functioning (learning difficulties and truancy). Further, the variables of school functioning were investigated using mean SPAI-C scores (continuous variable), (“SPAI-C score”), as predictor variables in the robust multiple Poisson regression model, involving the total sample who answered the self-report questionnaire (*n* = 8,199). The same was performed with a mean score of SCL-5 (“SCL-5 score”) as the predictor (*n* = 8,199). Results are reported as rate ratios (RR) along with 95% confidence intervals (95% CI).

Finally, multiple logistic regression analysis was performed to estimate the odds ratio (OR) for the association between the ADIS-C SP group and aspirations for further education, using the ADIS-C SN group as the reference group (*n* = 6,610). The same procedure was performed using mean scores of (1) SPAI-C and (2) SCL-5 as predictors, involving the total sample (*n* = 8,199). The analyses for SPAI-C and SCL-5 as predictors were then repeated and performed separately for students in lower secondary school (13–15 years of age) and upper secondary school (≥16 years of age). Results are reported as odds ratios (OR) with 95% confidence intervals (95% CI). All analyses were adjusted for sex, age (measured on a continuous scale), and perception of family economic status.

Lastly, in the published article, there was a minor error when describing descriptive characteristics and aspirations of going to university. We want to include “partly” to the sentence describing the tendency seen in SP subgroups. Even though the general tendency is also seen in the subgroups, we see the need for nuancing.

A correction has been made to *Results, Descriptive Characteristics, Paragraph 2*. The corrected paragraph is shown below.

A lower proportion of the SP group reported aspirations of going to university compared to the SN group (SP: 34.1%, SN: 39.2%), and a higher proportion of the SP group answered no plans/don't know (SP: 45.8%, SN: 41.8%). This was also partly observed for the three SP subgroups (see Supplementary Material). Regarding age differences, a higher proportion of adolescents under the age of 16 answered no plans/don't know (48.6%) compared to adolescents over the age of 16 (35.4%). For remaining descriptive characteristics, see Table 1.

In the published article, there was also an error in Supplementary Table 1. In the rows “Mean all social anxiety items (SPAI-C) (sd),” “Mean anxiety and depression items (SCL-5) (sd),” “Behavioral difficulties/attention problems (1-4),” “School dissatisfaction (1-4),” “Social exclusion/bullying (1-4),” “Truancy (single item) (1-4)” and “Learning difficulties (single item) (1-4),” the mean values were erroneously reported with standard errors (SE) instead of standard deviations (SD). The corrected Supplementary Table 1 and its caption appear below.

**Supplementary Table 1:** Descriptive characteristics of ADIS-C screening positive subgroups: screening positives that did not meet to interview (*n*=176), screening positives met to interview NOT diagnosed (*n*=106), and screening positives met to interview and diagnosed with SAD (*n*=106).

	**Screening positives that did not meet to interview**	**Screening positives met to interview NOT diagnosed**	**Screening positives met to interview and diagnosed with SAD**
**Sex n (%)**			
Girls	120 (68.18)	62 (58.49)	85 (80.19)
Boys	56 (31.82)	44 (41.51)	21 (19.81)
**Age mean (SD)**	16.47 (2.13)	15.90 (1.65)	15.74 (1.63)
**Age distribution n (%)**			
13-15 years	76 (43.18)	57 (53.77)	62 (58.49)
≥ 16 years	100 (56.82)	49 (46.23)	44 (41.51)
**Family economic status n (%)**			
Worse	29 (18.13)	11 (11.00)	17 (17.35)
Equal	109 (68.13)	68 (68.00)	73 (74.49)
Better	22 (13.75)	21 (21.00)	8 (8.16)
**Mean all social anxiety items (SPAI-C) (SD)**	2.86 (.86)	2.54 (.78)	3.04 (.92)
**Mean anxiety and depression items (SCL-5) (SD)**	2.05 (.73)	1.84 (.66)	2.11 (.74)
**Mean school functioning (SD)**			
Behavioral difficulties/attention problems (1-4)	1.72 (.44)	1.66 (.35)	1.75 (.42)
School dissatisfaction (1-4)	2.49 (.54)	2.37 (.53)	2.49 (.45)
Social exclusion/bullying (1-4)	1.51 (.68)	1.46 (.53)	1.64 (.76)
Truancy (1-4)	1.43 (.63)	1.27 (.47)	1.31 (.56)
Learning difficulties (1-4)	1.37 (.80)	1.40 (.83)	1.33 (.74)
**Educational aspirations n (%)**			
No plans/don't know	80 (49.69)	43 (43.88)	41 (41.41)
Vocational training	34 (21.12)	21 (21.43)	17 (17.17)
University	47 (29.19)	34 (34.69)	41 (41.41)

^*^Missing values: for family economic status values were missing for n=16/9.1% of the n=176 screening positives that did not meet to interview, n=6/5.7% of the n=106 screening positives met to interview and NOT diagnosed, and n=8/7.5% of the n=106 screening positives met to interview and diagnosed with SAD.

Mean score of SPAI-C had missing values for n=11/6.3%, n=4/3.8%, and n=4/3.8%. Mean score of SCL-5 had missing values for n=11/6.3%, n=3/2.8%, and n=2/1.9%.

For school functioning, mean scores of behavioral difficulties/attention problems had missing values for n=22/12.5%, n=9/8.5%, and n=9/8.5%, mean scores of school dissatisfaction: n=27/15.3%, n=10/9.4%, and n=9/8.5%, mean scores of social exclusion: n=20/11.4%, n=8/7.5%, and n=8/7.5%. For the single items, missing values were n=19/10.8%, n=6/5.7%, and n=8/7.5% (truancy), and n=27/15.3%, n=11/10.4%, and n=10/9.4% (learning difficulties).

For educational aspirations, variables were missing for n=15/8.5%, n=8/7.5%, and n=7/6.6%.

More detailed descriptions of the sample described in this table can be found in Jystad et al. (2021).

The authors apologize for these errors and state that they do not change the scientific conclusions of the article in any way. The original article has been updated.

