# Supplementary material for: School Functioning and Educational Aspirations in Adolescents With Social Anxiety—The Young-HUNT3 Study, Norway
Source: Front Psychol. 2021 Oct 12;12:727529. doi: 10.3389/fpsyg.2021.727529 (PMC8546336; doi:10.3389/fpsyg.2021.727529)
Supplement: Supplementary file 1 [file Data_Sheet_1.docx]

| **Supplementary Table 1.**  Descriptive characteristics of ADIS-C screening positive subgroups: screening positives that did not meet to interview (*n*=176), screening positives met to interview NOT diagnosed (*n*=106), and screening positives met to interview and diagnosed with SAD (*n*=106). | | | | |
| --- | --- | --- | --- | --- |
|  | | **Screening positives that did not meet to interview** | **Screening positives met to interview NOT diagnosed** | **Screening positives met to interview and diagnosed with SAD** |
| **Sex n (%)** | |  |  |  |
| Girls | | 120 (68.18) | 62 (58.49) | 85 (80.19) |
| Boys | | 56 (31.82) | 44 (41.51) | 21 (19.81) |
| **Age mean (sd)** | | 16.47 (2.13) | 15.90 (1.65) | 15.74 (1.63) |
| **Age distribution n (%)** | |  |  |  |
| 13-15 years | | 76 (43.18) | 57 (53.77) | 62 (58.49) |
| >=16 years | | 100 (56.82) | 49 (46.23) | 44 (41.51) |
| **Family economic status n (%)** | |  |  |  |
| Worse | | 29 (18.13) | 11 (11.00) | 17 (17.35) |
| Equal | | 109 (68.13) | 68 (68.00) | 73 (74.49) |
| Better | | 22 (13.75) | 21 (21.00) | 8 (8.16) |
| **Mean all social anxiety items (SPAI-C) (sd)** | | 2.86 (.86) | 2.54 (.78) | 3.04 (.92) |
| **Mean anxiety and depression items (SCL-5) (sd)** | | 2.05 (.73) | 1.84 (.66) | 2.11 (.74) |
| **Mean school functioning (sd)** | |  |  |  |
| Behavioral difficulties/attention problems (1-4) | | 1.72 (.44) | 1.66 (.35) | 1.75 (.42) |
| School dissatisfaction (1-4) | | 2.49 (.54) | 2.37 (.53) | 2.49 (.45) |
| Social exclusion/bullying (1-4) | | 1.51 (.68) | 1.47 (.53) | 1.64 (.76) |
| Truancy (1-4) | | 1.43 (.63) | 1.27 (.47) | 1.31 (.56) |
| Learning difficulties (0-4) | | 1.37 (.80) | 1.40 (.83) | 1.33 (.74) |
| **Educational aspirations n (%)** | |  |  |  |
| No plans/don’t know | | 80 (49.69) | 43 (43.88) | 41 (41.41) |
| Vocational training | | 34 (21.12) | 21 (21.43) | 17 (17.17) |
| University | | 47 (29.19) | 34 (34.69) | 41 (41.41) |
| *Missing values: for family economic status values were missing for n=16/9.1% of the n=176 screening positives that did not meet to interview, n=6/5.7% of the n=106 screening positives met to interview and NOT diagnosed, and n=8/7.5% of the n=106 screening positives met to interview and diagnosed with SAD.  Mean score of SPAI-C had missing values for n=11/6.3%, n=4/3.8%, and n=4/3.8%. Mean score of SCL-5 had missing values for n=11/6.3%, n=3/2.8%, and n=2/1.9%.  For school functioning, mean scores of behavioral difficulties/attention problems had missing values for n=22/12.5%, n=9/8.5%, and n=9/8.5%, mean scores of school dissatisfaction: n=27/15.3%, n=10/9.4%, and n=9/8.5%, mean scores of social exclusion: n=20/11.4%, n=8/7.5%, and n=8/7.5%. For the single items, missing values were n=19/10.8%, n=6/5.7%, and n=8/7.5% (truancy), and n=27/15.3%, n=11/10.4%, and n=10/9.4% (learning difficulties).  For educational aspirations, variables were missing for n=15/8.5%, n=8/7.5%, and n=7/6.6%.  More detailed descriptions of the sample described in this table can be found in Jystad et al. (2021). | | | | |
|  |  |  |  |  |

| \| **Supplementary Table 2.** Associations* between ADIS-C screening positive individuals and indicators of school functioning. Rate Ratio (RR) and 95 per cent confidence interval (95% CI). \| \| \| \| \| \| \| \| \| \| \| \| \| --- \| --- \| --- \| --- \| --- \| --- \| --- \| --- \| --- \| --- \| --- \| --- \| \|  \| **Behavioral difficulties/attention problems**  Range: 6-24 \| \| **School**  **dissatisfaction**  Range: 6-24 \| \| **Social**  **exclusion/bullying**  Range: 2-8 \| \| **Truancy/**  **school absenteeism**  Range: 1-4 \| \| \| **Learning problems**  Range: 1-4 \| \| \| *Screening status*** \| RR  (*n*=5983) \| 95% CI \| RR  (*n*=6003) \| 95% CI \| RR  (*n*=6074) \| 95% CI \| RR  (*n*=6131) \| 95% CI \| \| RR  (*n*=5887) \| 95% CI \| \| **ADIS-C SN** \| 1 (reference) \| \| 1 (reference) \| \| 1 (Reference) \| \| 1 (reference) \| \| \| 1 (reference) \| \| \| **ADIS-C SP not met to interview** \| **1.07** \| 1.03-1.11 \| **1.17** \| 1.13-1.21 \| **1.23** \| 1.15-1.32 \| **1.09** \| \| 1.02-1.16 \| **1.11** \| 1.01-1.22 \| \| **ADIS-C SP met to interview not diagnosed** \| 1.03 \| .99-1.08 \| **1.12** \| 1.07-1.17 \| **1.18** \| 1.10-1.27 \| 1.01 \| \| .95-1.08 \| 1.11 \| .98-1.25 \| \| **ADIS-C SP met to interview and diagnosed** \| **1.09** \| 1.04-1.15 \| **1.16** \| 1.12-1.20 \| **1.32** \| 1.20-1.44 \| 1.04 \| \| .96-1.13 \| 1.06 \| .95-1.18 \| \| **^*^**^)^ Adjusted for sex, age and family economic status.^**)^ Missing values: see table S1. \| \| \| \| \| \| \| \| \| \| \| \| |
| --- | --- | --- | --- | --- | --- | --- | --- | --- | --- | --- | --- | --- | --- | --- | --- | --- | --- | --- | --- | --- | --- | --- | --- | --- | --- | --- | --- | --- | --- | --- | --- | --- | --- | --- | --- | --- | --- | --- | --- | --- | --- | --- | --- | --- | --- | --- | --- | --- | --- | --- | --- | --- | --- | --- | --- | --- | --- | --- | --- | --- | --- | --- | --- | --- | --- | --- | --- | --- | --- | --- | --- | --- | --- | --- | --- | --- | --- | --- | --- | --- | --- | --- | --- | --- | --- | --- | --- | --- | --- | --- | --- | --- | --- | --- | --- | --- |

| **Supplementary Table 3** Associations* between SP subgroups (ADIS-C) and aspirations of higher education and aspirations for the future among adolescents. Odds ratio (OR) and 95 per cent confidence interval (95% CI). | | | | |
| --- | --- | --- | --- | --- |
|  |  | | | |
|  | **Aspirations of higher education** | | **Aspirations for the future** | |
| *Screening status*** | Adjusted OR  (*n*=5725) | 95% CI | Adjusted OR  (*n*=5725) | 95% CI |
| **ADIS-C screening negative** | 1 (Reference) |  | 1 (Reference) |  |
| **ADIS-C SP not met to interview** | **.56** | .39-.80 | **.70** | .50-.98 |
| **ADIS-C SP met to interview not diagnosed with SAD** | .77 | .50-1.20 | .92 | .60-1.39 |
| **ADIS-C SP met to interview and diagnosed with SAD** | 1.08 | .71-1.65 | 1.15 | .75-1.76 |
| ^*)^ Adjusted for sex, age and family economic status.  ^**)^ Missing values: see table S1. | | | | |
|  | | | | |
